# Blood Pressure and Cardio-Metabolic Risk Profile in Young Saudi Males in a University Setting

**DOI:** 10.3390/medicina57080755

**Published:** 2021-07-26

**Authors:** Said EL-Ashker, Mangesh S. Pednekar, Sameer S. Narake, Waleed Albaker, Mohammed Al-Hariri

**Affiliations:** 1 Self-Development Department, Deanship of Preparatory Year, Imam Abdulrahman Bin Faisal University, Dammam 31441, Saudi Arabia; sgelashker@iau.edu.sa; 2 Healis, Sekhsaria Institute for Public Health, Navi Mumbai 400701, India; pednekarm@healis.org (M.S.P.); narakes@healis.org (S.S.N.); 3 Department of Internal Medicine, College of Medicine, Imam Abdulrahman Bin Faisal University, Dammam 31451, Saudi Arabia; wialbakr@iau.edu.sa; 4 Department of Physiology, College of Medicine, Imam Abdulrahman Bin Faisal University, Dammam 31451, Saudi Arabia

**Keywords:** prehypertension, students, university, obesity, cardiovascular

## Abstract

*Background and Objectives*: The prevalence of cardiovascular diseases (CVDs) poses significant clinical and public health challenges across the world. This study aimed to study the metabolic risk factors and the association with blood pressure alteration. *Materials and Methods*: This was a cross-sectional study conducted between 2017 and 2018 among 284 male university students in Eastern province, Saudi Arabia. The obesity and cardiovascular measurements were taken using standardized instruments, including blood pressure (BP), mean arterial pressure, body mass index (BMI), body adiposity index (BAI), waist circumference (WC), waist-to-hip ratio (WHR), waist-to-height ratio (WHtR), body fat percentage (BFP), and basal metabolic rate (BMR). Statistical Analysis: Blood pressure was classified according to the United States of America, Sixth Joint National committee (JNC-VI) guidelines. The mean and standard error were calculated for each hypertension group variable. Logistic regression was applied to predict associations. *Results:* The prevalence of hypertension in the present study was 61.6%., and that of overweight and obesity was 16.5% and 34.9%, respectively. The cut-off values of BMI and WC were 22.23 and 75.24, respectively. *Conclusions*: The results demonstrated that BMI, WC, WHR, and WHtR significantly predict hypertension and that WC has a greater discrimination capacity than other measures. The findings also emphasize the importance of cardiovascular risk screening for young adults to detect any alterations in blood pressure and thus identify the population that is vulnerable to CVDs at an early stage. The findings highlight the need for health and university policymakers to adopt measures to monitor and control hypertension and obesity at the university level.

## 1. Introduction

Obesity is a major risk factor for a group of diseases and death. It is a well-established cardio-metabolic risk factor causing excess adiposity and cardiovascular diseases (CVDs) [1]. Obesity is a chronic disorder due to abnormal excessive fat accumulation and may have negative health outcomes [2]. The World Health Organization stated that, globally, among adults aged 18 years or more, 39% were overweight and 13% were obese [3]. A community-based national epidemiological health survey conducted in Saudi Arabia examined 17,232 adults aged 30 to 70 years and reported 39.9% (females 31.8%, males 42.4%) as overweight and 35.5% (female 44%, 26.4% males) as obese [4]. Among the other CVD risk factors, high blood pressure accounts for the greatest proportion of global deaths [5]. Hypertension is an essential risk factor for many health conditions, including stroke and coronary heart disease. It is also well reported that obese patients are more likely to be hypertensive than nonobese patients [6]. Although little scientific information is available, the pathophysiological changes of hypertension show that blood pressure alteration is linked to adiposity [1].

Epidemiological reports of the relationship between blood pressure (BP) alterations and adiposity commonly use anthropometric measurements, including height, weight, hip circumference (HC), and waist circumference (WC). Indices of obesity such as the waist-to-height ratio (WHtR), waist-hip ratio (WHR), and body mass index (BMI) can be derived from these measurements [7]. Obesity has been classified according to BMI levels: “BMI between 25 and 29.9 is considered to be overweight, and a BMI ≥ 30 is considered to be obese” [8,9]. Among these measurements, it has long been recognized that BMI (kg/m^2^) and WC are significant predictors of mortality and morbidity associated with multiple disorders, such as CVD, stroke, and type 2 diabetes [10].

The number of deaths due to CVD is estimated to reach 23.3 million globally by the year 2030. However, CVD can be reduced or controlled by tackling the predisposing risk factors, such as unhealthy diet, physical inactivity, obesity, diabetes mellitus, and use of tobacco [11]. Furthermore, in countries including Saudi Arabia there is an increasing burden of adiposity-related diseases such as CVD due to various demographic, socioeconomic, and health transitions [6,12]. According to a recent report, the prevalence of hypertension among citizens in Saudi Arabia was 11.1%, and the prevalence of overweight and obesity together was 69.9% [13]. This denotes a population that is highly vulnerable to experiencing morbidity and mortality due to CVD in the near future.

Primary prevention measures, with a focus on decreasing avoidable risk factors such as high blood pressure and body weight, may be the most effective approach for the prevention and control of CVD [14]. Prehypertension is defined as the transient stage between normal and high blood pressure (120–139/80–89 mmHg) and a risk factor for developing hypertension and target organs damage. Importantly, prehypertension is controllable, especially in young obese people, and is often correlated with other cardiometabolic risk factors [15].

A study conducted in 2015 among university students reported 7.5% as hypertensive. Among the participants, 51.6% had a normal BMI, 29.8% were overweight, 10.7% were moderately obese, and 7.9% were severely obese [16].

In recent years, scarce research has been undertaken to estimate the prevalence of obesity and prehypertension among university students. Moreover, data on prehypertension in adults is not steady due to the adopted lifestyle characteristics in Saudi Arabia. It is also essential to understand the predisposing risk factors in this vulnerable group of individuals so that directed and channelized health measures can be provided to them.

The early diagnosis of individuals who have prehypertension is important in order to control the blood pressure and avoid or minimize any potential complications. Therefore, the aim of the present study was to assess the prevalence of prehypertension and to identify risk factors associated with BP changes among male university students in Eastern province, Saudi Arabia.

## 2. Materials and Methods

### 2.1. Study Design and Setting

A cross-sectional study was conducted between 2017 and 2018 in Eastern Province, Saudi Arabia among male university students aged 17–24 years. All male students (*N* = 1241) were invited to participate. Only 352 students responded and agreed to participate. Sixty-eight students from the 352 were excluded because they did not attend the measurement sessions or because they reported having some chronic diseases or any other disabilities that might have affected their anthropometric measures. Therefore, 284 students completed all of the intended measures and were available for this analysis. The ethical approval for the study was obtained by the local Ethics Committee and Review Board at the Imam Abdulrahman Bin Faisal University, Saudi Arabia (approval; IRB-2018-19-112/19-4-2018).

### 2.2. Data Collection Tools

The cardiovascular and obesity measurements were obtained by qualified research assistants according to international standards and references [17]. The measurements were taken using standardized equipment. Participants were asked to wear light clothing and remove their shoes before taking their measurements. The height of the students was documented to the nearest 0.1 cm, and the weight was calculated to the nearest 0.1 kg (Seca 704; Seca, Hamburg, Germany) [18]. The measurements of WC and WHR are two of the most commonly used noninvasive biomarkers in predicting cardio-metabolic risk factors. WC was measured with a measuring tape (Gay Mill, WI, USA) to the nearest centimeter (cm) midway between the inferior angle of the ribs and the suprailiac crest. The HC was measured as the maximal circumference over the buttocks in centimeters [19].

Two BP records were taken (after 5 min of rest) using an automatic BP monitor (Omron M6 Comfort IT), and if there was a difference in the two records a third was recorded [20,21]. Measurements were taken for all subjects around midday, i.e., 11.00–12.30 h. To ensure the greatest reliability of the gathered data, every student was requested to press the dynamometer two times with a one minute rest between each recording. The mean of the BP recordings was considered for the data analysis [22].

### 2.3. Indices’ Calculations

Based on the blood pressure measurements, the students were classified for hypertension under the following categories: normal (<120 systolic and/or <80 mmHg diastolic); prehypertension (120–139/80–89); hypertension stage 1 (140–159/90–99); and hypertension stage 2 (≥160/≥100) [23]. Based on the BMI measurements, the students were categorized as underweight (<18.5), normal weight (18.5–24.9), overweight (25.0–29.9), and obese (30+) [24]. Furthermore, the cardiovascular and obesity indices were calculated accordingly, as shown in Table 1.

### 2.4. Statistical Analysis

Statistical analyses were performed using the SPSS 20.0 software package (IBM, Chicago, IL, USA). The mean and standard errors were calculated for each study group (normal, prehypertension, hypertension (stage 1 and stage 2)) for all the measurement variables. A bivariate logistic regression was applied to predict the association between the blood pressure and anthropometric measurement variables. Two dependent variables were created as (1) normal blood pressure = 0 vs. prehypertension = 1, and (2) normal blood pressure = 0 vs. stage 1 or 2 hypertension = 2. The anthropometric measurement variables were kept as independent continuous variables while running the logistic regression. The receiver operating characteristic (ROC) test was undertaken to determine the cut-off values of cardiovascular and obesity indices to identify the risk of pre- and/or hypertension [30,31]. The specificity and sensitivity were analyzed, and the point having the highest sum was taken as the cut-off value for the indicator. *p* values < 0.05 were considered to be statistically significant.

## 3. Results

The mean and standard error (S.E) for the anthropometric measurement variables for each hypertension group are depicted in Table 2. The prevalence (proportion) of prehypertension was found to be 61.6%, and that of stage 1 and stage 2 was found to be 12% and 2.8%, respectively. The mean values for heart rate, pulse pressure, MAP, WHR, WC, BAI, BMR, BMI, and BFP were comparatively higher among the prehypertension or hypertension groups than the normal individuals. Table 3 shows the odds ratios for the anthropometric predictor variables for the prehypertension and hypertension (stage 1 or stage 2) groups. There was an association of heart rate, pulse pressure, MAP, weight, HC, WC, BAI, BMR, BMI, and BFP with the occurrence of prehypertension or hypertension. The association of mean arterial pressure was stronger (OR = 1.94 CI 1.60, 2.37 and OR = 1.72 CI 1.29, 2.30) than the other measured variables.

Table 4 shows the crude odds ratios for hypertension in comparison, with reference to the normal BMI. The risk is significantly higher among overweight (OR = 7.12, CI 2.40, 21.14) and obese (OR = 32.11, CI 7.55, 136.7) students. The prevalence of overweight and obesity was found to be 16.5% and 34.9% based on the categories defined by the WHO as a BMI between 25 to 29 and a BMI ≥ 30, respectively. A share of 38.7% of the students had higher WC than cut-off values (92), and 30.6% of the students had a higher WHC ratio than cut-off values (0.89).

**Figure 1:**
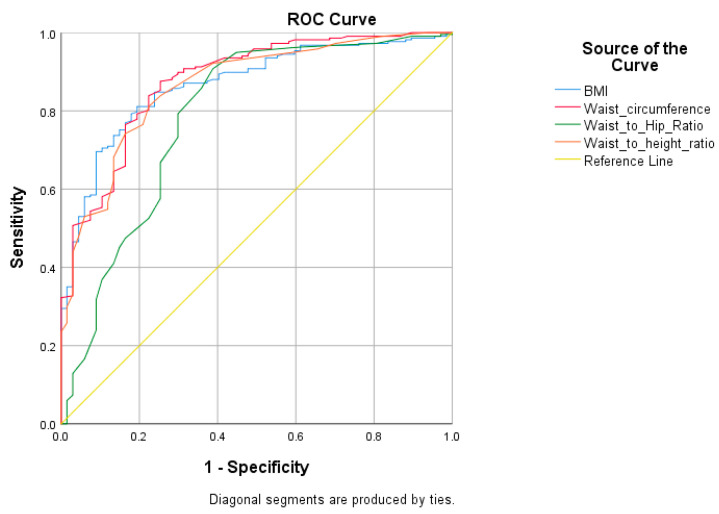
ROC curve for four metabolic predicator variables for hypertension.

The cut-off values were calculated by a ROC curve analysis to identify the risk of hypertension for various measurements. The cut-off value of BMI for the study population was 22.23 (specificity = 0.806, sensitivity = 0.811), and that of WC was 75.24 (sensitivity = 0.876, specificity = 0.746), as presented in Table 5. The WC (AUC (0.878)) shows a greater discriminating capacity than others (Table 6). Figure 1 shows that the ROC curve plot for BMI, WC, WHR, and WHtR significantly predicts hypertension.

## 4. Discussion

In our study, the prevalence of prehypertension among students was very high. Obesity indices were associated with prehypertension among university students in the Eastern Province of Saudi Arabia. However, the OR for hypertension was highly dependent on the obesity measurements when the logistic regression model was used. Moreover, the multivariate logistic regression analysis (adjusted only for age) showed that the prevalence of prehypertension increased in the study participants with the increase in WC in contrast to hypertension. The results are consistent with another local study conducted in Alkharj (a governorate in Saudi Arabia) in which the prevalence of prehypertension was 66.1%, with 48% in the male age group of 18–30 years [32].

The results from both the ROC curve and logistic regression analyses showed that the “strongest” predictor for hypertension in the study sample was WC. Numerous researchers have found that WC was more strongly associated with cardiovascular morbidity and mortality than other measurements [30,33]. More recently, a recommendation was given to consider WC as an important “clinical indicator” to evaluate the central or visceral fat [33]. Similarly, reported studies have found that central obesity calculated by WC is more strongly associated with the risk of incident cardiovascular events than BMI [1,34].

As documented, the accumulation of body fat increases morbidity and mortality related to different health disorders including pre- and/or hypertension. Our findings are consistent with previous study results wherein body fat accumulation was significantly associated with prehypertension and correlated with the male sex and those who were urban citizens [35,36]. The present data showed that more than half of the students were overweight (16.5%) and obese (34.9%). When compared to another study conducted among the same population, the prevalence of obesity among the young generation in Saudi Arabia has increased dramatically [37]. The study also suggests that the current sedentary lifestyle prevalent in this age group is responsible for the high incidence of obesity. In addition, several previous reports showed that playing videos games, watching television, and the prolonged use of computers are playing a vital role in the propagation of this unhealthy lifestyle epidemic [38,39].

Furthermore, this age group is highly significant because these students join universities to gain their higher education and should therefore apply their maximum efforts and time to their studies. In addition, the time used for outdoor games and physical activities is limited due to their studies [40]. Sedentary lifestyles are highly associated with obesity and, unfortunately, young students do not consider that such a lifestyle is predisposed to cardiovascular morbidity and mortality [18,41]. Generally, among the young generation, obese people are 5.94 times more likely to have hypertension than their normal-weight counterparts [42].

Although the etiopathogenesis of obesity-associated hypertension via “renin-angiotensin-aldosterone system activation, increased secretion of leptin, insulin resistance, sympathetic nervous system stimulation and other biochemical active compounds” has been explained in previous reports, the pathophysiological mechanisms underlying obesity-related outcomes, including hypertension and other metabolic health disorders, are still unclear and need further studies. Thus, early identification and recognition of the early risks of cardiovascular diseases such as hypertension will help in the decision making for possible preventive and predictive strategies [43].

## 5. Conclusions

In conclusion, the present results demonstrated an alarming rate of increase in the prevalence of prehypertension and obesity among university students in the Eastern Providence of Saudi Arabia. It also reflects the dissemination of the sedentary lifestyle among university students. Furthermore, our findings emphasize the importance of cardiovascular risk screening for young adults in order to detect any alterations in BP at the earliest possible stage. The results highlight that the prevalence of hypertension and/or prehypertension increased significantly with the increase in the obesity indicators among the study population. Therefore, health and university policymakers must consider the predisposing factors among this vulnerable population in order to prepare preventive and health promotion strategies for controlling CVD.

### Limitation

The limitations of the present study are its cross-sectional nature, the use of only male participants, and the lack of adjustment for appropriate confounders.

## Figures and Tables

**Table 1:** Cardiovascular and obesity indices calculation.

Parameter	Formula	Reference
Mean arterial pressure	MAP = diastolic blood pressure (DBP) + 1/3 [systolic blood pressure (SBP) − DBP].	[25]
Body adiposity index	BAI = (Hip circumference [HC] in centimeters)/((height in meters)1.5) − 18).	[26]
Waist-to-Hip Ratio	WHR = Waist circumference (WC) in centimeters ÷ HC in centimeters.	[27]
Body Mass Index (kg/m^2^)	BMI = (weight in kilograms)/(the squared height in meters).	[24]
Body fat percentage	BFP = (1.20 × BMI) + (0.23 × age) − (10.8 × sex) − 5.4, (sex: females = 0, males = 1).	[28]
Basal Metabolic Rate (kcal/day)	Men BMR = 66.5 + (13.8 × weight) + (5.0 × height) − (6.8 × age).	[29]

kcal/day = kilocalories per day.

**Table 2:** Characteristics of participants by hypertension group.

	*N*	*p* Value for Trend	Hypertension Group								Total (*n* = 284)	
			Normal (*n* = 67)		Prehypertension (*n* = 175)		Stage-1 (*n* = 34)		Stage-2 (*n* = 8)			
			Mean	S.E.	Mean	S.E.	Mean	S.E.	Mean	S.E.	Mean	S.E.
Age (years)	284	0.181	18.5	0.1	18.7	0.1	18.6	0.2	19.3	0.3	18.6	0.1
Heart Rate (BPM)	284	0.001 *	71.9	1.5	80.0	0.6	90.2	2.9	100.3	5.5	79.9	0.7
Pulse pressure (mmHg)	284	0.003 *	45.8	0.6	55.5	0.5	67.3	2.2	81.3	8.9	55.4	0.7
Mean arterial pressure	284	0.008 *	80.6	0.5	90.6	0.3	101.9	1.1	119.0	7.8	90.4	0.6
Height (m)	284	0.367	1.7	0.0	1.7	0.0	1.7	0.0	1.7	0.0	1.7	0.0
Weight (kg)	284	0.035 *	59.4	1.2	82.7	1.7	119.2	5.3	123.5	14.2	82.7	1.7
Hip circumference (cm)	284	0.030 *	90.2	1.1	107.3	1.0	124.7	2.8	128.4	8.4	105.9	1.0
Waist circumference (cm)	284	0.016 *	72.2	1.3	91.5	1.2	113.5	3.4	121.2	12.5	90.4	1.2
Body adiposity index	284	0.014 *	23.2	0.5	30.2	0.5	36.4	1.2	39.4	3.8	29.6	0.4
Waist-to-Hip Ratio	284	0.008 *	0.8	0.0	0.8	0.0	0.9	0.0	0.9	0.1	0.8	0.0
BMI (kg/m^2^)	284	0.020 *	20.9	0.4	28.4	0.6	39.3	1.6	42.2	4.9	28.3	0.6
Body fat percentage	284	0.020 *	23.9	0.5	33.0	0.7	46.1	2.0	49.7	5.9	32.9	0.7
Basal Metabolic Rate (kcal/day)	284	0.042 *	1561.9	13.0	1805.6	17.4	2190.0	54.9	2213.4	147.2	1805.6	17.6

BPM = beats per minute; BMI = body mass index; kcal/day = kilocalorie per day; * = statistically significant *p* value (*p* < 0.05).

**Table 3:** Association between the study measurements and hypertension.

	Normal vs. Prehypertension			Normal vs. Hypertension (Stage I or II)		
	OR	95% C.I.		OR	95% C.I.	
		Lower	Upper		Lower	Upper
Age	1.25	0.93	1.69	1.36	0.86	2.15
Heart Rate	1.09	1.06	1.13	1.10	1.06	1.14
Pulse pressure	1.37	1.26	1.49	1.33	1.18	1.49
Mean arterial pressure	1.94	1.60	2.37	1.72	1.29	2.30
Weight	1.13	1.09	1.18	1.12	1.07	1.17
Hip circumference	1.18	1.12	1.24	1.18	1.11	1.26
Waist circumference	1.14	1.10	1.19	1.12	1.08	1.16
Body adiposity index	1.30	1.20	1.42	1.40	1.24	1.58
BMI	1.37	1.24	1.53	1.37	1.21	1.55
Body fat percentage	1.31	1.20	1.43	1.30	1.17	1.44
Basal Metabolic Rate	1.01	1.01	1.01	1.01	1.01	1.02

OR = Odds Ratio, C.I. = Confidence Interval.

**Table 4:** The prevalence of overweight/obesity and odds ratio for hypertension (prehypertension, or stage 1 and stage 2) compared to normal blood pressure.

Anthropometric Indicator	*n* (%)	Crude OR (95% CI)	Age Adjusted OR (95% CI)
Body mass index (kg/m^2^)			
<18.5	20 (7.0)	0.28 (0.10, 0.79)	0.21 (0.07, 0.63)
18.5–24.9	118 (41.5)	1	1
25.0–29.9	47 (16.5)	7.12 (2.40, 21.14)	6.85 (2.28, 2.55)
30+	99 (34.9)	32.11 (7.55, 136.57)	32.26 (7.54, 137.99)
Waist circumference (cm)			
<cut-off (92)	174 (61.3)	1	1
≥cut-off	110 (38.7)	32.20 (7.69, 134.83)	35.26 (8.36, 148.68)
Waist-hip circumference ratio			
<cut-off (0.89)	197 (69.4)	1	1
≥cut-off	87 (30.6)	5.01 (2.18, 11.48)	5.19 (2.24, 12.0)
Waist-height ratio			
<0.5	263 (92.6)	1	1
≥0.5	21 (7.4)	*	*

* = cell frequency was 0 for normal BP category, hence ORs cannot be estimated; OR = odds ratio, C.I. = confidence interval.

**Table 5:** Optimal cut-off values for different metabolic indices, and their sensitivities and specificities for hypertension.

	Cut Off	Sensitivity	Specificity
BMI (kg/m^2^)	22.23	0.811	0.806
Waist circumference (cm)	75.24	0.876	0.746
Waist-hip circumference ratio	0.785	0.908	0.092
Waist-height ratio	0.455	0.811	0.776

**Table 6:** Area under receiver operating characteristic curves of metabolic indices for screening hypertension.

Test Result Variable(s)	Area	Std. Error ^a^	Asymptotic Sig.^b^	Asymptotic 95% Confidence Interval	
				Lower Bound	Upper Bound
Body Mass Index	0.868	0.023	<0.001	0.823	0.914
Waist circumference	0.878	0.024	<0.001	0.831	0.924
Waist-to-Hip Ratio	0.787	0.036	<0.001	0.716	0.858
Waist-to-height ratio	0.865	0.025	<0.001	0.817	0.913

Note: ^a^. Under the nonparametric assumption, ^b^. Null hypothesis: true area = 0.5.

## Data Availability

The data will be available upon reasonable request.
